# Association of childhood obesity with risk of early all-cause and cause-specific mortality: A Swedish prospective cohort study

**DOI:** 10.1371/journal.pmed.1003078

**Published:** 2020-03-18

**Authors:** Louise Lindberg, Pernilla Danielsson, Martina Persson, Claude Marcus, Emilia Hagman

**Affiliations:** 1 Division of Pediatrics, Department of Clinical Science, Intervention and Technology, Karolinska Institutet, Stockholm, Sweden; 2 Clinical Epidemiology Division, Department of Medicine, Solna, Karolinska Institutet, Stockholm, Sweden; 3 Department of Diabetes and Endocrinology, Sachsska Children’s Hospital, Södersjukhuset, Stockholm, Sweden; 4 Department of Clinical Science and Education, Södersjukhuset, Karolinska Institutet, Stockholm, Sweden; London School of Hygiene and Tropical Medicine, UNITED KINGDOM

## Abstract

**Background:**

Pediatric obesity is associated with increased risk of premature death from middle age onward, but whether the risk is already increased in young adulthood is unclear. The aim was to investigate whether individuals who had obesity in childhood have an increased mortality risk in young adulthood, compared with a population-based comparison group.

**Methods and findings:**

In this prospective cohort study, we linked nationwide registers and collected data on 41,359 individuals. Individuals enrolled at age 3–17.9 years in the Swedish Childhood Obesity Treatment Register (BORIS) and living in Sweden on their 18th birthday (start of follow-up) were included. A comparison group was matched by year of birth, sex, and area of residence. We analyzed all-cause mortality and cause-specific mortality using Cox proportional hazards models, adjusted according to group, sex, Nordic origin, and parental socioeconomic status (SES). Over 190,752 person-years of follow-up (median follow-up time 3.6 years), 104 deaths were recorded. Median (IQR) age at death was 22.0 (20.0–24.5) years. In the childhood obesity cohort, 0.55% (*n =* 39) died during the follow-up period, compared to 0.19% (*n =* 65) in the comparison group (*p* < 0.001). More than a quarter of the deaths among individuals in the childhood obesity cohort had obesity recorded as a primary or contributing cause of death. Male sex and low parental SES were associated with premature all-cause mortality. Suicide and self-harm with undetermined intent were the main cause of death in both groups. The largest difference between the groups lay within endogenous causes of death, where children who had undergone obesity treatment had an adjusted mortality rate ratio of 4.04 (95% CI 2.00–8.17, *p* < 0.001) compared with the comparison group. The main study limitation was the lack of anthropometric data in the comparison group.

**Conclusions:**

Our study shows that the risk of mortality in early adulthood may be higher for individuals who had obesity in childhood compared to a population-based comparison group.

## Abstract 2

Louise Lindberg et al demonstrate association of paediatric obesity with mortality in young adulthood using the Swedish Childhood Obesity Treatment Register (BORIS).

## Author summary

**Why was this study done?:**

- Obesity in childhood and adolescence is a global public health concern.
- It has been shown that childhood obesity increases the risk of death from middle age onward, but whether obesity in childhood increases the risk of premature death in young adulthood is unknown.

**What did the researchers do and find?:**

- We followed 41,359 individuals into young adulthood, of whom 7,049 had been offered obesity treatment in childhood, and investigated the risk of mortality.
- This study demonstrates that individuals who had obesity in childhood had a 3 times higher risk of mortality in early adulthood compared with a population-based comparison group. Both the risk of death due to diseases and the risk of death due to suicide were higher among those who had obesity in childhood, but no increased risk of mortality due to injuries or external causes was observed.

**What do these findings mean?:**

- Obesity in childhood and adolescence is a highly prevalent and severe condition with increased risks of associated morbidity and premature mortality. Our results further support the importance of continued efforts to provide psychosocial support and improve treatment for obesity in childhood and adolescence.

## Introduction

Obesity in childhood is a global public health concern and one of the largest challenges of the 21st century. The prevalence of obesity among children and adolescents has increased around the world [1], and it has been estimated that 91 million children will have obesity in 2025 [2]. Obesity in childhood is associated with somatic morbidity such as insulin resistance, liver disease, and hypertension [3–6]. Further, children with obesity may also experience emotional and psychological problems [7–9] and are often exposed to bullying by their peers [10,11]. Moreover, obesity in childhood and adolescence often persists into adulthood [8,12,13], with high rates of comorbidity [14] and social exclusion [15–17]. Overweight and obesity under 18 years of age has also been linked to an increased risk of premature mortality from middle adulthood onward [18]. A limited number of studies, conducted before the obesity epidemic, with baseline data collected during the period 1940–1975, have investigated the association between measured BMI in adolescence and risk of mortality in young adulthood [12,19–22]. While 1 study found a correlation between adolescent BMI and mortality risk before the age of 30 years [19], 2 of the studies did not find such an association [20,22]. High BMI in young adulthood has been associated with a higher risk of premature death later in life, largely driven by cardiovascular disease and other noncancer diseases [23–25]. To our knowledge, there are no current studies on risk of mortality in young adulthood in relation to measured height and weight in childhood. Therefore, the aim was to examine whether individuals who had obesity in childhood have an increased risk of mortality in young adulthood, compared with a population-based comparison group.

## Methods

### Individuals

Individuals were included if they were enrolled in the Swedish Childhood Obesity Treatment Register (BORIS) at age 3–17.9 years, and alive and living in Sweden on their 18th birthday (start of follow-up, *n = *7,049). No exclusion criteria were applied. BORIS was initiated in 2005 and is a prospective register of children and adolescents in obesity treatment [26]. A description of BORIS can be found elsewhere [26], but, in short, data are entered into the register by clinicians at the healthcare units treating obesity. The aim of the register is to support all aspects of decision-making around childhood obesity such as evaluation of different treatment regimens and administrative actions, and thereby advance the quality and cost-effectiveness of obesity treatment. BORIS is integrated in the clinical workflows and generates data in real time.

Using a personal identification number unique to each resident in Sweden, a comparison group from the Total Population Register was historically (year of entrance in BORIS) matched with a ratio of 1:5 by sex, year of birth, and area of residence (*n =* 34,310). Individuals in BORIS were not eligible for inclusion in the comparison group. Density matching without replacement was used in the matching procedure (the RANUNI procedure in SAS was used to randomly select individuals to the comparison group). Among individuals in the comparison group, 1.3% had a diagnosis of obesity before 18 years of age from inpatient or outpatient care. All medical data in the present study were collected within the healthcare system; hence, no self-reported data are present.

The main exposure was obesity in childhood, defined using the International Obesity Task Force sex- and age-adjusted cutoffs for body mass index standard deviation score (BMI SDS) [27]. However, BMI SDS should be considered as a rough measurement since it does not correspond to the same fat percentage in different individuals.

### Data sources and register linkage

Information on date of death and primary cause and contributing causes of death was retrieved from the Cause of Death Register. The register has had complete coverage since 1997 and includes deaths that have occurred abroad. The completeness and quality of the register has been described elsewhere, and it has been concluded that the register is of high quality with largely complete national coverage [28]. Information on weight, height, and age at initiation of pediatric obesity treatment was retrieved from BORIS [26,29]. Information on parental education, occupation, and income was collected from the Longitudinal Integration Database for Health Insurance and Labour Market Studies. Information on country of birth and emigration was retrieved from the Swedish Total Population Register [30].

In order to be able to exclude individuals in sensitivity analyses, those with a diagnosis of a genetic syndrome and/or malignant tumor (including malignant and benign brain tumors) were identified in the National Patient Register using the International Classification of Diseases [31]. Genetic syndromes included fragile X, Klinefelter, Laurence–Moon–Bardet–Biedl, Down, Noonan, Prader–Willi, Silver–Russell, and Turner.

The National Board of Health and Welfare (https://www.socialstyrelsen.se/en) and Statistics Sweden (https://www.scb.se/en), both governmental agencies, are responsible for all the national registries mentioned above, except for BORIS. The study was approved by the regional ethics committee in Stockholm, Sweden (No. 2016/922-31/1).

This study is part of a larger epidemiological analysis using data from several national population-based registers. Before starting the analyses, a specific study plan was outlined stating which analyses would be performed. No specific data-driven changes were made; existing evidence was used for the study, i.e., data from national registers, and existing cutoffs and thresholds, e.g., for BMI SDS, were applied.

### All-cause mortality and cause-specific mortality

Mortality was assessed using all-cause mortality and cause-specific mortality. Follow-up began at 18 years of age and ended at the date of death, date of emigration, or end of follow-up (December 31, 2017), whichever came first. Mortality was defined as any death that occurred within the specified time period. Cause-specific mortality was categorized into 3 groups: endogenous causes, suicide and self-harm, and injuries and other external causes. Endogenous causes included death from pathogens, acquired disorders, congenital disorders, etc. Suicide and self-harm included intentional death from suicide as well as death with unintentional or unclear intention from poisoning, e.g., illicit drugs. Injuries and other external causes included deaths such as traffic accidents and homicide.

### Parental socioeconomic status and covariates

Parental socioeconomic status (SES) was estimated based on parental education, occupation, and income, assessed the year the child turned 16 years of age. Using the International Standard Classification of Education, education was categorized as elementary, high school, or university (score 0–2). Occupation was categorized as no occupation (score 0) or occupation (score 1). Annual disposable income was divided into quartiles based on the income of parents in the comparison group (score 0–3). The annual disposable income was converted using the Swedish Consumer Price Index to 2015 prices. The mean maternal and paternal SES score was calculated and divided into 4 categories: low SES (0–1.5 points), medium-low SES (2–3 points), medium-high SES (3.5–4.5 points), and high SES (5–6 points). If an individual was adopted, data on adoptive parents was used (childhood obesity cohort, *n =* 94; comparison group, *n =* 609).

Covariates included were sex, Nordic origin, and age and BMI SDS at start of obesity treatment. Nordic origin was defined as Nordic (the individual and at least 1 parent being born in a Nordic country [Sweden, Finland, Norway, Denmark, or Iceland]) or non-Nordic (the individual born outside the Nordic countries, or born in a Nordic country but with neither parent born in a Nordic country).

### Statistical analyses

Descriptive statistics are presented as frequency and percentage, mean and standard deviation (SD), or median and interquartile range (IQR). Power analyses were performed using the score test for Cox proportional hazards regression. To examine if the risk of all-cause mortality differed between groups, crude and adjusted Cox proportional hazards models were used to calculate mortality rate ratios (MRRs) and 95% confidence intervals (CIs). Adjusted models were controlled according to group (childhood obesity cohort or comparison group), sex, Nordic origin, and parental SES. The above analyses were repeated with cause-specific mortality (deaths with unknown cause were not included; childhood obesity cohort, *n =* 1; comparison group, *n =* 3). Kaplan–Meier analysis was used to investigate whether there was a difference in crude probability of survival between the groups. As post hoc analyses, sensitivity analyses were performed excluding individuals with genetic syndromes and malignant tumors in childhood. Also, degree of obesity (BMI SDS) was evaluated in the childhood obesity cohort. As parental SES was missing for only a limited number of individuals (childhood obesity cohort, 0.4% [*n =* 28]; comparison group, 0.9% [*n =* 308]), complete case analyses were done.

In analyses that included only individuals in the childhood obesity cohort, the potential impact of obesity severity (BMI SDS) and age at obesity treatment initiation was investigated. Analyses were performed with SAS version 9.4 (Cary, NC, US). A *p*-value of <0.05 was considered statistically significant.

## Results

In total, 41,359 individuals were included. Since the groups were matched on sex and age, there was an even distribution between groups with respect to sex (46% females) and age at end of follow-up (median age 21.6 years, IQR 19.6–24.7, maximum age 38.8 years). Individuals who had been referred to obesity treatment in childhood were followed on average 9.5 (SD 4.0) years from start of treatment to end of follow-up (maximum 23.0 years). Further, they were more likely to be of non-Nordic origin and to have low parental SES, genetic syndromes, and tumors before 18 years of age than individuals from the comparison group. Sample characteristics are shown in Table 1, and data stratified by sex are shown in S1 Table.

**Table 1:** Characteristics of the participants (*n* = 41,359).

Characteristic	Childhood obesity cohort	Comparison group	*p*-Value
Individuals	7,049 (100.0)	34,310 (100.0)	
Deaths	39 (0.55)	65 (0.19)	<0.001
Person-years of follow-up	32,501	158,251	
Nordic origin	5,119 (72.6)	25,327 (73.8)	0.038
Emigrated at ≥18 years of age	80 (1.1)	594 (1.7)	<0.001
Genetic syndrome^a^	65 (0.92)	90 (0.26)	<0.001
Malignant tumor <18 years of age^b^	60 (0.85)	107 (0.31)	<0.001
Parental SES			<0.001
Low	1,610 (22.8)	1,610 (15.3)	
Medium-low	2,672 (37.9)	10,863 (31.7)	
Medium-high	2,066 (29.3)	11,776 (34.3)	
High	673 (9.6)	6,106 (17.8)	
Missing	28 (0.4)	308 (0.9)	

Data are *n* (%) unless otherwise indicated.

^a^Fragile X, Klinefelter, Laurence–Moon–Bardet–Biedl, Down, Noonan, Prader–Willi, Silver–Russell, and Turner.

^b^Including benign brain tumors.

SES, socioeconomic status.

### All-cause mortality

During a total of 190,752 person-years of follow-up, 104 deaths were recorded. The median (IQR) age at death was 22.0 (20.0–24.5) years. Thirty-nine deaths (0.55%) occurred among the 7,049 individuals included in the childhood obesity cohort, corresponding to a mortality rate of 12.0 per 10,000 person-years. Sixty-five deaths (0.19%) occurred among the 34,310 individuals included in the comparison group, corresponding to a mortality rate of 4.1 per 10,000 person-years (*p* < 0.001). Among the deceased individuals, there was no difference between groups regarding sex (*p =* 0.37), age at death (*p =* 0.11), Nordic origin (*p =* 0.73), or parental SES (*p =* 0.84). In a reverse power calculation for the outcome all-cause mortality, the calculated power reached over 99%.

**Fig 1:**
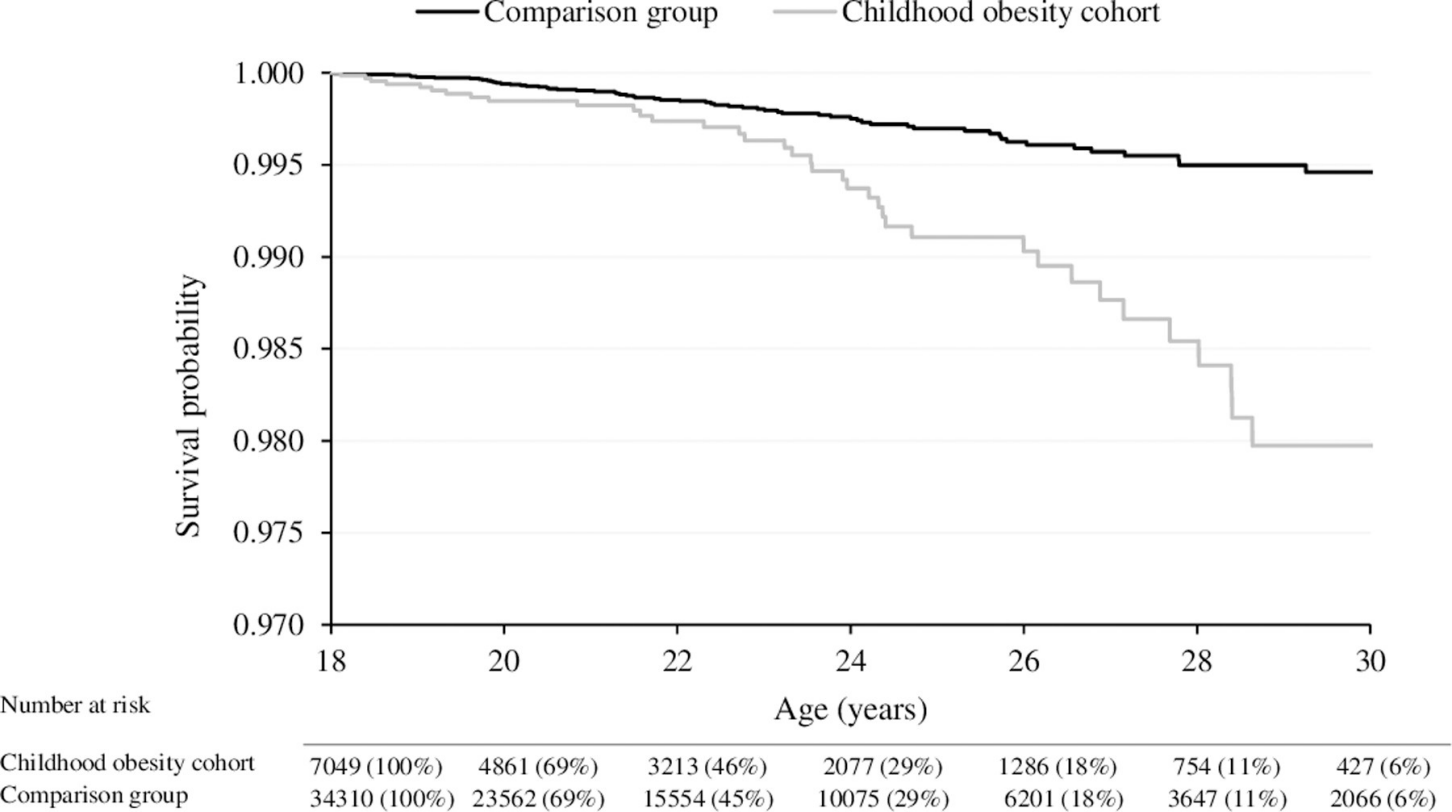
Survival curves of all-cause mortality in the childhood obesity cohort and the comparison group.

All-cause mortality was overall higher for individuals in the childhood obesity cohort compared to the comparison group (Fig 1). The probability of survival decreased with age, and the difference between the groups particularly increased from 23 years of age onward (Fig 1). Individuals in the childhood obesity cohort had an almost 3 times greater risk of all-cause mortality compared to individuals in the comparison group (crude MRR 2.92 [95% CI 1.97–4.35]; *p* < 0.001).

Analyzing both groups together, risk factors for all-cause mortality included male sex (crude MRR 1.74 [95% CI 1.17–2.60]; *p =* 0.007) and low parental SES (reference high SES, crude MRR 2.85 [95% CI 1.26–6.47]; *p =* 0.012), while Nordic origin was not associated with the outcome (*p =* 0.76). In analyses mutually adjusted according to group (childhood obesity cohort versus comparison group), sex, Nordic origin, and parental SES, the results were only mildly attenuated, and remained statistically significant (Table 2).

**Table 2:** Mutually adjusted mortality rate ratios (MRRs) with 95% confidence intervals (CIs) for all-cause mortality (*n =* 41,023).

	MRR	95% CI	*p*-Value
Group (childhood obesity cohort versus comparison group)	2.65	1.78–3.96	<0.001
Sex (male versus female)	1.75	1.17–2.62	0.006
Nordic origin (Nordic versus non-Nordic)	1.18	0.74–1.88	0.50
Parental SES			
Low versus high	2.58	1.11–5.99	0.028
Medium-low versus high	1.82	0.81–4.06	0.15
Medium-high versus high	1.11	0.47–2.63	0.81

Adjusted for group, sex, Nordic origin, and parental socioeconomic status (SES).

In sensitivity analyses, individuals with genetic syndromes (*n =* 155) and malignant tumors (*n =* 167) in childhood were excluded (childhood obesity cohort, 1.77%; comparison group, 0.57%). Of the excluded individuals, 10 died during the follow-up period. Individuals in the childhood obesity cohort were still at significantly higher risk of premature death compared to the comparison group (adjusted sensitivity analysis MRR 2.56 [95% CI 1.67–3.92]; *p* < 0.001).

### Cause-specific mortality

Information on cause-specific mortality was available for 97% (*n =* 38) of the deceased individuals in the childhood obesity cohort and 95% (*n =* 62) of the deceased individuals in the comparison group. Individuals who had undergone obesity treatment in childhood had an increased risk of death from suicide and self-harm and death from endogenous causes, compared to the comparison group (Table 3).

**Table 3:** Cause-specific mortality rate ratio (MMR) for the childhood obesity cohort compared to the comparison group.

Cause of death	Number of deaths		Mortality rate per 10,000 person-years	Crude MRR (95% CI); *p*-value	Adjusted MRR (95% CI); *p*-value
	Childhood obesity cohort	Comparison group			
Injuries and external causes	7	14	1.10	2.44 (0.98–6.04); 0.054	2.38 (0.96–5.94); 0.063
Endogenous causes	15	17	1.68	4.30 (2.15–8.61); <0.001	4.04 (2.00–8.17); <0.001
Suicide and self-harm	16	31	2.46	2.51 (1.38–4.60); 0.003	2.15 (1.17–3.95); 0.014
Excluding individuals with genetic syndromes^a^ and malignant tumors^b^ in childhood					
Injuries and external causes	7	14	1.33	2.49 (1.01–6.17); 0.049	2.43 (0.97–6.06); 0.057
Endogenous causes	11	12	1.46	4.56 (2.01–10.33); <0.001	4.50 (2.00–10.32); <0.001
Suicide and self-harm	15	31	2.92	2.40 (1.30–4.45); 0.005	2.03 (1.09–3.78); 0.026

Crude and adjusted models yielded MRRs for childhood obesity cohort versus comparison group; adjusted model controlled for sex, Nordic origin, and parental SES. Endogenous causes of death included deaths from pathogens, acquired disorders, congenital disorders, etc. Suicide and self-harm included intentional deaths from suicide as well as deaths with unintentional or unclear intention from poisoning, e.g., illicit drugs. Injuries and other external causes included deaths such as traffic accidents and homicide.

^a^Fragile X, Klinefelter, Laurence–Moon–Bardet–Biedl, Down, Noonan, Prader–Willi, Silver–Russell, and Turner.

^b^Including benign brain tumors.

Suicide and self-harm were the most common cause of death in both groups (Table 3) and primarily included death from poisoning, followed by suicide by hanging or jumping. Death due to endogenous causes showed the most pronounced difference in the childhood obesity cohort compared to the comparison group, with an MRR of 4.30 (Table 3). Overall, cancer was the most common endogenous cause of death, followed by infection. Among individuals in the childhood obesity cohort, 26% had obesity (ICD-10 code E66) as either primary or contributing cause of death, whereas in the comparison group, none had obesity as a cause of death. There was no statistically significant difference between individuals with and without obesity in risk of death from injuries and other external causes (MRR 2.38 [95% CI 0.96–5.94]; *p =* 0.063). However, the pattern was similar to that of endogenous causes and suicide and self-harm, with increased risk of injury or external cause of death in the childhood obesity cohort (Table 3). Transportation accidents, followed by homicide, were the most common causes of deaths from injuries and other external causes. A detailed list of primary causes of death is presented in S2 Table, where it can be observed, for example, that the proportion of individuals dying from cancer in the childhood obesity cohort was not greater than that in the comparison group.

The results remained in sensitivity analyses where individuals with genetic syndromes and malignant tumors in childhood were excluded (Table 3).

### Pediatric-obesity-related factors and all-cause mortality

Mean (SD) age at start of obesity treatment was 14.0 (2.3) years among the deceased individuals and 13.1 years (2.7) among the non-deceased individuals (*p =* 0.02). BMI SDS at start of obesity treatment was 3.3 (0.5) and 2.9 (0.5) BMI SDS units among the deceased and non-deceased individuals, respectively (*p* < 0.001). In analyses adjusted for age at start of obesity treatment, Nordic origin, sex, and parental SES, the severity of obesity at the start of treatment was associated with premature death (MRR per 0.5-unit increase in BMI SDS 1.79 [95% CI 1.29–2.48]; *p =* 0.001). Age at start of obesity treatment did not influence the outcome (crude MRR 1.00 [95% CI 0.88–1.14]; *p =* 0.97). At the last registered clinical visit, 81% of the individuals in the childhood obesity cohort still had obesity.

## Discussion

It is known that obesity increases the risk of premature death from middle age onward, but whether obesity in childhood increases the risk of premature death in young adulthood has, to our knowledge, not previously been studied. We followed children and adolescents identified in a pediatric obesity treatment register and a population-based comparison group into young adulthood to investigate risk of premature mortality. This study found that individuals who had obesity in childhood had a 3 times higher risk of all-cause mortality in early adulthood compared with a population-based comparison group. The result was only slightly attenuated after adjusting for sex, Nordic origin, and parental SES.

Suicide and self-harm were the most common cause of death in both groups. The largest difference in cause-specific death between the groups was for endogenous causes, where the MRR was 4 times higher in the childhood obesity cohort compared to the comparison group. Furthermore, 1 in 4 deaths among individuals who had obesity in childhood had obesity recorded as a primary or contributing cause of death.

### Comparisons to previous studies

In the present study the mortality rate in the childhood obesity cohort was 12.0 per 10,000 person-years, while the expected mortality rate (from the comparison group) was 4.1 deaths per 10,000 person-years. Previous estimated risks of premature death in young adulthood are based on data collected roughly 50–70 years ago [12,19–22], before the obesity epidemic, and used different cutoffs for obesity [19,20] than the currently recommended one (≥30 kg/m^2^) [32]. It is unclear to what extent the population with obesity more than half a century ago is representative of the population of children with obesity today. Further, during these previous study periods, obesity was uncommon, and only a small percentage of the individuals had obesity in adolescence [20–22]. This makes comparisons between studies problematic.

In the current study, being of non-Nordic origin did not predict risk of all-cause mortality in early adulthood. In contrast, a US-based study, unadjusted for BMI and tracking deaths over a period of 15 years, showed associations between mortality and different ethnic groups, demonstrating a higher mortality risk among ethnic minorities [33]. We can only speculate about reasons for these conflicting findings, but they might include different definitions of ethnicity and differences in healthcare systems between countries. Furthermore, we found that males had an increased risk of all-cause mortality compared to females, which is consistent with previous research examining 20- to 30-year-old adults with obesity [23].

SES affects health throughout life [34,35]. Family conditions, such as parental working status and education, that determine SES in early life are associated with mortality risk in adulthood [36,37], and may also influence long-term morbidity [35,38]. Whether these risks are irrespective of individuals’ own SES in adulthood is inconclusive [36,37]. In the current study, low parental SES, compared to high, was associated with risk of all-cause mortality in young adulthood almost to the same extent as pediatric obesity. As pediatric obesity is more common in families with limited socioeconomic resources, as also seen in the present study, one may keep in mind that children with obesity may be burdened by several risk factors.

The present study found differences in cause-specific mortality between the groups. The risk of death from endogenous causes was especially increased in the childhood obesity cohort. Previous studies have shown a correlation between adolescent BMI and risk of premature death in middle age from cardiovascular disease [24,39], ischemic heart disease, and endocrine, nutritional, and metabolic diseases [21]. Several forms of cancer have also been associated with obesity in adults [40]. However, in our study we did not see a significant influence of cancer during follow-up on observed mortality in the childhood obesity cohort compared with the comparison group, perhaps due to the young age within the study sample. We also identified an increased risk of death from suicide and self-harm for individuals with obesity in childhood compared to the comparison group. This is in line with some [39], but not all [21], previous studies.

### Children and adolescents with obesity and premature death

The severity of obesity at the start of obesity treatment was a risk factor for premature death. This has, to our knowledge, not been demonstrated previously. However, in a study including almost 5,000 individuals (Native Americans born 1945–1984, aged 5 to <20 years, almost 30% with obesity), those in the highest quartile of BMI, compared to the lowest, had a significantly greater risk of death from endogenous, but not external, causes [41].

The association between obesity and risk of premature mortality could be explained by several mediating factors of both somatic and non-somatic origin. Obesity in childhood has been linked to systemic low-grade inflammation [42], non-alcoholic fatty liver disease [43], insulin resistance [43], and impaired cardiovascular health, including thicker intima media thickness [44], elevated blood pressure [43], and impaired microvascular function [45]. Further, overweight and obesity have been associated with depression [7,9], discrimination, and bullying [11]. The design of the present study does not allow conclusions on causality to be drawn regarding obesity in childhood and mortality in young adulthood. However, both somatic and psychological factors may play a role in the increased risk of mortality observed in individuals with obesity. Although it is known that successful obesity treatment in childhood, based on behavioral lifestyle modification including diet and physical activity, reduces risk factors that may influence risk of mortality [4,17,46], studies investigating effective obesity treatments in children and their impact on mortality risk itself are lacking. Due to the relatively limited number of deaths occurring in early adulthood, we were not able to examine the effect of obesity treatment response on risk of mortality. However, it is well-established that weight loss has positive long-term health benefits [4,17,46], and has in adults been shown to reduce the risk of premature mortality [25]. Future research should investigate whether successful obesity treatment can lead to a reduction in the risk of early mortality.

### Limitations

Despite the many relationships between obesity and severe morbidities [47], it has been questioned whether it is possible to study associations between BMI and mortality under the age of 30 years because of the very low mortality rate [22]. A reverse power analysis showed that our study had a large enough study sample for the actual number of events. It has also been argued that a long follow-up time is necessary to investigate deaths due to illness influenced by BMI [22]. However, even as early as childhood and adolescence, obesity has been linked to several obesity-related risk factors, as previously discussed. In the current study, 40% of all deaths were due to endogenous causes, and of all deaths with cause recorded, a quarter were related to obesity. Still, a larger population, longer follow-up, and medical data in adulthood would allow more complex statistics, including stratification by sex, evaluation of whether the association is different across ages, and evaluation of whether obesity treatment outcome affects the outcome.

Another limitation of the present study may be that we did not apply any exclusion criteria, e.g., genetic syndromes. The prevalence of such conditions among the pediatric obesity population may proportionally have become lower over the years due to the obesity epidemic, but individuals with such conditions are still an important part of the patient group. However, we did perform sensitivity analyses where individuals with conditions potentially associated with premature mortality were excluded.

Weight and height were measured for all individuals in the childhood obesity cohort; however, these data were not available in the comparison group. Obesity in adolescence often persists into adulthood [12]. Over 80% of individuals with obesity at their first clinical visit in the present study still had obesity at their last available clinical visit. Thus, although we lack follow-up measures of weight and height in young adulthood, it is reasonable to assume that the majority still had obesity at the end of follow-up.

Further, it is possible that the relationship between obesity in childhood and mortality risk in early adulthood is confounded by unmeasured factors, e.g., risk behaviors such as smoking and alcohol consumption. However, studies have shown that adjusting for smoking status does not modify the mortality risks in individuals with obesity [12,48,49].

Four of the deceased individuals had an unknown cause of death and were thus not included in the cause-specific analyses. The reasons for these missing data might include declining rates of autopsy, or death abroad with inability to determine cause of death. Nevertheless, overall, the Cause of Death Register is a high-quality, virtually complete register of all deaths in Sweden since 1952 and contains both primary and contributing causes of death, indicating a potential chain that led to death [28].

## Conclusion

This prospective cohort study shows that individuals who had obesity in childhood already have an increased risk of death by early adulthood, compared with a population-based comparison group. Among those who had obesity in childhood, 1 in 4 had obesity recorded as a cause of death. Identifying specific factors that may impact the risk of early mortality in individuals with obesity in childhood is important, to enable preventive actions and to promote long-term health.

## Supporting information

S1 STROBE Checklist Strengthening the reporting of observational studies in epidemiology (STROBE) checklist. (PDF)

S1 Table Characteristics of the participants (*n =* 41,359) stratified by sex. (PDF)

S2 Table Primary cause-specific mortality. (PDF)

S1 Text English translation of relevant parts of the study analysis plan and general methods. (PDF)

S2 Text Full study analysis plan in Swedish. (PDF)

## Abbreviations

BMI SDS: body mass index standard deviation score
BORIS: Swedish Childhood Obesity Treatment Register
MRR: mortality rate ratio
SES: socioeconomic status
