# Supplementary material for: Association of childhood obesity with risk of early all-cause and cause-specific mortality: A Swedish prospective cohort study
Source: PLoS Med. 2020 Mar 18;17(3):e1003078. doi: 10.1371/journal.pmed.1003078 (PMC7080224; doi:10.1371/journal.pmed.1003078)
Supplement: S1 STROBE Checklist — (PDF) [file pmed.1003078.s001.pdf]

STROBE Statement—checklist of items that should be included in reports of observational studies

	Item No.	Recommendation	Author comment	Relevant text from manuscript
**Title and abstract**	1	(*a*) Indicate the study's design with a commonly used term in the title or the abstract	Stated in the title and in the abstract	"Association of childhood obesity with risk of early all-cause and cause-specific mortality: a Swedish prospective cohort study"<br><br>"In this prospective cohort study…"
		(*b*) Provide in the abstract an informative and balanced summary of what was done and what was found	Stated in the abstract	"The aim was to investigate whether individuals who had obesity in childhood, have an increased mortality risk in young adulthood, compared with a population-based comparison group."<br><br>**"**. Individuals enrolled at age 3–17.9 years in the Swedish Childhood Obesity Treatment Register (BORIS) and living in Sweden on their 18th birthday (start of follow-up) were included. A comparison group was matched by year of birth, sex, and area of residence."<br><br>**"**In the childhood obesity cohort, 0.55% (n=39) died during the follow-up period, compared to 0.19% (n=65) in the comparison group (p<0.0001).**"**
**Introduction**				
Background/rationale	2	Explain the scientific background and rationale for the investigation being reported	Stated in the introduction	"Overweight and obesity under 18 years of age has also been linked to an increased risk of premature mortality from middle adulthood onward. A limited number of studies, conducted before the obesity epidemic, with baseline data collected during the period 1940-1975, have investigated the association between measured BMI in adolescence and risk of mortality in young adulthood."<br><br>"To our knowledge, there are no current studies on risk of mortality in young adulthood in relation to measured height and weight in childhood."
Objectives	3	State specific objectives, including any prespecified hypotheses	Introduction, last sentence	"Therefore, the aim was to examine whether individuals who had obesity in childhood have an increased risk of mortality in young adulthood, compared with a population-based comparison group."

**Methods**				
Study design	4	Present key elements of study design early in the paper	Methods, paragraph 1-2	"Individuals were included if they were enrolled in the Swedish Childhood Obesity Treatment Register (BORIS) at age 3–17.9 years, and alive and living in Sweden on their 18th birthday (start of follow-up, *n* = 7,049)."<br><br>"Using a personal identification number, unique to each resident in Sweden, a comparison group from the Total Population Register was historically (year of entrance in BORIS) matched …"
Setting	5	Describe the setting, locations, and relevant dates, including periods of recruitment, exposure, follow-up, and data collection	Methods, paragraph 1 & 6,	"Individuals were included if they were enrolled in the Swedish Childhood Obesity Treatment Register (BORIS) at age 3–17.9 years, and alive and living in Sweden on their 18th birthday (start of follow-up, *n* = 7,049)."<br><br>"Follow-up began at 18 years of age and ended at date of death, date of emigration, or end of follow-up (December 31st 2017), whichever came first."
Participants	6	(*a*) *Cohort study*—Give the eligibility criteria, and the sources and methods of selection of participants. Describe methods of follow-up<br>*Case-control study*—Give the eligibility criteria, and the sources and methods of case ascertainment and control selection. Give the rationale for the choice of cases and controls<br>*Cross-sectional study*—Give the eligibility criteria, and the sources and methods of selection of participants	Methods, paragraph 1 & 6	"Individuals were included if they were enrolled in the Swedish Childhood Obesity Treatment Register (BORIS) at age 3–17.9 years, and alive and living in Sweden on their 18th birthday (start of follow-up, *n* = 7,049)."<br><br>"No exclusion criteria were applied."<br><br>"Follow-up began at 18 years of age and ended at the date of death, date of emigration, or end of follow-up (December 31st 2017), whichever came first."
		(*b*) *Cohort study*—For matched studies, give matching criteria and number of exposed and unexposed<br>*Case-control study*—For matched studies, give matching criteria and the number of controls per case	Methods, paragraph 1-2	"Individuals were included if they were registered in the Swedish Childhood Obesity Treatment Register (BORIS)…(n=7,049)."<br><br>"…a comparison group from the Total Population Register was historically (year of entrance in BORIS) matched with a ratio of 1:5 by sex, year of birth, and area of residence (n=34,310). Individuals in BORIS were not eligible for inclusion in the comparison group. Density matching without replacement was used in the matching procedure…"

Variables	7	Clearly define all outcomes, exposures, predictors, potential confounders, and effect modifiers. Give diagnostic criteria, if applicable	Methods, paragraph 2, 4, & 6-8	"The main exposure was obesity in childhood, defined using the International Obesity Task Force sex- and age-adjusted cut-offs for body mass index standard deviation score (BMI SDS)."<br><br>"Mortality was assessed using all-cause mortality and cause-specific mortality."<br><br>" Cause-specific mortality was categorized into three groups;…"<br><br>"Parental socioeconomic status (SES) was estimated based on parental education, occupation, and income,…"<br><br>"Covariates included were sex, Nordic origin, and age and BMI SDS at start of obesity treatment."<br><br>"…those with a diagnosis of genetic syndromes and/or malignant tumors (including malignant and benign brain tumors) were identified… "Genetic syndromes included Fragile X, Klinefelter, Laurence-Moon-Bardet-Biedl, Down, Noonan, Prader-Willi, Silver Russell, and Turner."
Data sources/ measurement	8*	For each variable of interest, give sources of data and details of methods of assessment (measurement). Describe comparability of assessment methods if there is more than one group	Methods, paragraph 3-4	"Information on date of death and primary cause and contributing causes of death was retrieved from the Cause of Death Register."<br><br>"Information on weight, height, and age at initiation of pediatric obesity treatment was retrieved from BORIS. Information on parental education, occupation, and income was collected from the Longitudinal Integration Database for Health Insurance and Labour Market Studies. Information on country of birth and emigration was retrieved from the Swedish Total Population Register"<br><br>"…a diagnosis of a genetic syndrome and/or malignant tumors (including malignant and benign brain tumors) were identified in the National Patient Register…"
Bias	9	Describe any efforts to address potential sources of bias	Methods, paragraph 2 & 5	"Using a personal identification number, unique to each resident in Sweden, a comparison group from the Total Population Register was historically (year of entrance in BORIS) matched …"

				"The National Board for Health and Welfare (www.socialstyrelsen.se/en) and Statistics Sweden (www.scb.se/en), both governmental agencies, are responsible for all the national registries mentioned above…"
Study size	10	Explain how the study size was arrived at	Methods, paragraph 1-2	"Individuals were included if they were enrolled in the Swedish Childhood Obesity Treatment Register (BORIS) at age 3–17.9 years, and alive and living in Sweden on their 18th birthday (start of follow-up, *n* = 7,049). No exclusion criteria were applied."<br><br>"a comparison group from the Total Population Register was historically (year of entrance in BORIS) matched with a ratio of 1:5 by sex, year of birth, and area of residence (n=34,310)."
Quantitative variables	11	Explain how quantitative variables were handled in the analyses. If applicable, describe which groupings were chosen and why	Methods, paragraph 3 & 7	"Information on weight, height, and age at initiation of pediatric obesity treatment was retrieved from BORIS"<br><br>"The mean maternal and paternal SES score was calculated and divided into four categories; low SES (0-1.5 points), medium-low SES (2-3 points), medium-high SES (3.5-4.5 points), and high SES (5-6 points)."
Statistical methods	12	(*a*) Describe all statistical methods, including those used to control for confounding	Methods, paragraph 9	"Power analyses were performed using the score test for Cox proportional hazards regression. To examine if the risk of all-cause mortality differed between groups, crude and adjusted Cox proportional hazard models were used to calculate mortality rate ratios (MRRs) and 95% confidence intervals (CIs). Adjusted models were controlled according to…"<br><br>"Kaplan-Meier analysis was used to investigate whether there was a difference in crude probability of survival between the groups."
		(*b*) Describe any methods used to examine subgroups and interactions	Methods, paragraph 10	"In analyses including individuals in the childhood obesity cohort only, the potential impact of obesity severity (BMI SDS) and age at obesity treatment initiation was investigated."
		(*c*) Explain how missing data were addressed	Methods, paragraph 9	"As parental SES was missing for only a limited number of individuals (childhood obesity cohort, 0.4% [*n* = 28]; comparison group, 0.9% [*n* = 308]), complete case analyses were done."
		(*d*) *Cohort study*—If applicable, explain how loss to follow-up was addressed		N/A

		Case-control study—If applicable, explain how matching of cases and controls was addressed<br>*Cross-sectional study*—If applicable, describe analytical methods taking account of sampling strategy		
		(*e*) Describe any sensitivity analyses	Methods, paragraph 9	"As post hoc analyses, sensitivity analyses were performed excluding individuals with genetic syndromes and malignant tumors in childhood."
**Results**				
Participants	13*	(a) Report numbers of individuals at each stage of study—eg numbers potentially eligible, examined for eligibility, confirmed eligible, included in the study, completing follow-up, and analysed	Results, paragraph 1 & 6	"In total 41,359 individuals were included."<br><br>"Information on cause-specific mortality was available for 97% (n=38) of the deceased in the childhood obesity cohort and 95% (n=62) of the deceased in the comparison group."
		(b) Give reasons for non-participation at each stage	Methods, paragraph 9 and Discussion paragraph 13	"As parental SES was missing for only a limited number of individuals (childhood obesity cohort, 0.4% [*n* = 28]; comparison group, 0.9% [*n* = 308]), complete case analyses were done."<br><br>"Four of the deceased had an unknown cause of death and were thus not included in the cause-specific analyses. The reasons for this might include declining rates of autopsy, or death abroad with inability to determine cause of death."
		(c) Consider use of a flow diagram		
Descriptive data	14*	(a) Give characteristics of study participants (eg demographic, clinical, social) and information on exposures and potential confounders	Results, paragraph 1, Table 1 and S1 Table	"Since the groups were matched on sex and age, there was an even distribution between groups with respect to sex (46% females) and age at end of follow-up (median age 21.6 years, IQR 19.6-24.7, maximum age 38.8 years)."<br><br>"Further, they were more likely to be of non-Nordic origin, have low parental SES, genetic syndromes, and tumors before 18 years of age than individuals from the comparison group."
		(b) Indicate number of participants with missing data for each variable of interest	Methods, paragraph 9	"As parental SES was missing for only a limited number of individuals (childhood obesity cohort, 0.4% [*n* = 28]; comparison group, 0.9% [*n* = 308]), complete case analyses were done."

		(c) *Cohort study*—Summarise follow-up time (eg, average and total amount)	Results, paragraph 1-2	"Individuals who had been referred to obesity treatment in childhood were followed on average 9.5 (SD 4.0) years from start of treatment to end of follow-up (maximum 23.0 years)."<br><br>"During a total of 190,752 person-years of follow-up,…"
Outcome data	15*	*Cohort study*—Report numbers of outcome events or summary measures over time	Results, paragraph 2 & 6	"Thirty-nine deaths (0.55%) occurred among the 7,049 individuals included in the childhood obesity cohort... Sixty-five deaths (0.19%) occurred among the 34,310 individuals included in the comparison group..."<br><br>"Information on cause-specific mortality was available for 97% (*n = 38*) of the deceased individuals in the childhood obesity cohort and 95% (*n* = 62) of the deceased individuals in the comparison group."
		Case-control study—Report numbers in each exposure category, or summary measures of exposure		N/A
		Cross-sectional study—Report numbers of outcome events or summary measures		N/A
Main results	16	(*a*) Give unadjusted estimates and, if applicable, confounder-adjusted estimates and their precision (eg, 95% confidence interval). Make clear which confounders were adjusted for and why they were included	Results, paragraph 3, 4, & 7<br>Fig 1, Table 2 & Table 3	"All-cause mortality was overall higher for individuals in the childhood obesity cohort compared to the comparison group (Fig 1)"<br><br>"Individuals in the childhood obesity cohort had an almost three times greater risk of all-cause mortality compared to individuals in the comparison group..."<br><br>"In analyses mutually adjusted according to group (childhood obesity cohort vs. comparison group), sex, Nordic origin, and parental SES, the results were only mildly attenuated, and remained statistically significant (Table 2)."<br><br>"Suicide and self-harm were the most common cause of death in both groups (Table 3)…"<br><br>"Death due to endogenous causes showed the most pronounced difference in the childhood obesity cohort compared to the comparison group…"
		(*b*) Report category boundaries when continuous variables were categorized	Methods, paragraph 7	"The mean maternal and paternal SES score was calculated and divided into four categories; low SES (0-1.5 points), medium-low SES (2-3 points), medium-high SES (3.5-4.5 points), and high SES (5-6 points)."

		(*c*) If relevant, consider translating estimates of relative risk into absolute risk for a meaningful time period		
Other analyses	17	Report other analyses done—eg analyses of subgroups and interactions, and sensitivity analyses	Results, paragraph 5 & 8	"In sensitivity analyses, individuals with genetic syndromes (n=155) and malignant tumors (n=167) in childhood were excluded (childhood obesity cohort=1.77%, comparison group=0.57%)."<br><br>"In analyses adjusted for age at start of obesity treatment, Nordic origin, sex, and parental SES, the severity of obesity at the start of treatment was associated with premature death (MRR per 0.5-unit increase in BMI SDS 1.79 [95% CI 1.29–2.48]; p = 0.001). Age at start of obesity treatment did not influence the outcome..."
**Discussion**				
Key results	18	Summarise key results with reference to study objectives	Discussion, paragraph 1-2	"This study found that individuals who had obesity in childhood had a 3 times higher risk of all-cause mortality in early adulthood compared with a population-based comparison group."<br><br>"Suicide and self-harm were the most common cause of death in both groups. The largest difference in cause-specific death between the groups was for endogenous causes, where the MRR was 4 times higher in the childhood obesity cohort compared to the comparison group. Furthermore, 1 in 4 deaths among individuals who had obesity in childhood had obesity recorded as a primary or contributing cause of death."
Limitations	19	Discuss limitations of the study, taking into account sources of potential bias or imprecision. Discuss both direction and magnitude of any potential bias	Discussion, paragraph 9-13	"…it has been questioned whether it is possible to study associations between BMI and mortality under the age of 30 years because of the very low mortality rate."<br><br>"…we did not apply any exclusion criteria, e.g. genetics syndromes."<br><br>"Weight and height were measured for all individuals in the obesity cohort, however, these data were not available in the comparison group."<br><br>"…it is possible that the relationship between obesity in childhood and mortality risk in early adulthood is confounded by unmeasured factors,…"

				"Four of the deceased individuals had an unknown cause of death and were thus not included in the cause-specific analyses."
Interpretation	20	Give a cautious overall interpretation of results considering objectives, limitations, multiplicity of analyses, results from similar studies, and other relevant evidence	Discussion, paragraph 3-8	"In the present study the mortality rate in the childhood obesity cohort was 12.0 per 10,000 person-years, while the expected mortality rate (from the comparison group) was 4.1 deaths per 10,000 person-years. Previous estimated risks of premature death in young adulthood are based on data collected roughly 50-70 years ago…"<br><br>"…being of non-Nordic origin did not predict risk of all-cause mortality in early adulthood. In contrast, a U.S. based study, …showed associations between mortality and different ethnic groups, demonstrating a higher mortality risk among ethnic minorities"<br><br>"Family conditions, such as parental working status and education, …are associated with mortality risk in adulthood… In the current study, low parental SES, compared to high, was associated with risk of all-cause mortality in young adulthood almost to the same extent as pediatric obesity."<br><br>"Several forms of cancer have also been associated with obesity in adults. However, in our study we did not see a significant influence of cancer during follow-up on the observed mortality..."<br><br>"The severity of obesity at the start of obesity treatment was a risk factor for premature death. This has to our knowledge not been demonstrated previously."<br><br>"The association between obesity and risk of premature mortality could be explained by several mediating factors of both somatic and non-somatic origin..."<br><br>"…overweight and obesity have been associated with depression, discrimination, and bullying …both somatic and psychological factors may play a role in the increased risk of mortality observed in individuals with obesity."
Generalisability	21	Discuss the generalisability (external validity) of the study results	Discussion, paragraph 9 & 13	"Despite the many relationships between obesity and severe morbidities, it has been questioned whether it is possible to study associations between BMI and mortality under the age of 30 years because of the very low mortality rate. A reverse power analysis showed that our study had a large enough study sample for

			the actual number of events. It has also been argued that a long follow-up time is necessary to investigate deaths due to illness influenced by BMI.”<br><br>“…the Cause of Death Register is a high-quality, virtually complete register of all deaths in Sweden since 1952 and contains both primary and contributing causes of death, indicating a potential chain that led to death.”
**Other information**			
Funding	22	Give the source of funding and the role of the funders for the present study and, if applicable, for the original study on which the present article is based	“This study was supported by grants from: The Swedish Heart and Lung Foundation, 20150790, C.M. https://www.hjart-lungfonden.se/; The Crown Princess Lovisa’s Foundation, 4-1727/2018, L.L. http://www.kronprinsessanlovisa.se/; Sällskapet barnavård, 4-1562/2018, L.L. https://ki.se/kbh/sallskapet-barnavard; Mjölkdroppen Foundation, 4-1200/2019, L.L. http://www.mjolkdroppen.se/; Fredrik and Ingrid Thurings Foundation, 2018-00385, E.H. http://www.thuringsstiftelse.com/; Magnus Bergvalls Foundation, 2018-02962, E.H. http://www.magnbergvallsstiftelse.nu/. In addition, L.L. has personal grants from The Samariten Foundation, 2018-0412, https://stiftelsensamariten.se/; The Sven Jerring Foundation, https://jerringfonden.se/; Solstickan Foundation https://solstickan.se/. The funders had no role in study design, data collection and analysis, decision to publish, or preparation of the manuscript.”

*Give information separately for cases and controls in case-control studies and, if applicable, for exposed and unexposed groups in cohort and cross-sectional studies.

Note: An Explanation and Elaboration article discusses each checklist item and gives methodological background and published examples of transparent reporting. The STROBE checklist is best used in conjunction with this article (freely available on the Web sites of PLoS Medicine at http://www.plosmedicine.org/, Annals of Internal Medicine at http://www.annals.org/, and Epidemiology at http://www.epidem.com/). Information on the STROBE Initiative is available at www.strobe-statement.org.
