# Supplementary material for: Association of childhood obesity with risk of early all-cause and cause-specific mortality: A Swedish prospective cohort study
Source: PLoS Med. 2020 Mar 18;17(3):e1003078. doi: 10.1371/journal.pmed.1003078 (PMC7080224; doi:10.1371/journal.pmed.1003078)
Supplement: S1 Table — (PDF) [file pmed.1003078.s002.pdf]

S1 Table. Characteristics of the participants (n = 41,359) stratified by sex.

	Girls			Boys		
**Characteristic**	**Childhood obesity cohort**	**Comparison group**	***p*-Value**	**Childhood obesity cohort**	**Comparison group**	***p*-Value**
Individuals	3,231 (100.0)	15,734 (100.0)		3,818 (100.0)	18,576 (100.0)	
Deaths	16 (0.50)	21 (0.13)	<.001	23 (0.60)	44 (0.24)	<0.001
Person-years of follow-up	15,684	76,418		16,817	81,833	
Nordic origin	2,362 (73.1)	11,539 (73.3)	0.78	2,757 (72.2)	13,788 (74.2)	0.0099
Emigrated at ≥18 years of age	46 (1.4)	327 (2.1)	0.015	34 (0.9)	267 (1.4)	0.0075
Genetic syndrome[a]	27 (0.84)	36 (0.23)	<.001	38 (1.00)	54 (0.29)	<.001
Malignant tumor <18 years of age[b]	34 (1.01)	42 (0.27)	<.001	26 (0.68)	65 (0.35)	0.0031
Parental SES			<.001			<.001
Low	771 (23.9)	2,456 (15.6)		839 (22.0)	2,801 (15.1)	
Medium-low	1,228 (38.0)	4,977 (31.6)		1,444 (37.8)	5,886 (31.7)	
Medium-high	927 (28.7)	5,434 (34.5)		1,139 (29.8)	6,342 (34.1)	
High	288 (8.9)	2,746 (17.5)		385 (10.1)	3,360 (18.1)	
Missing	17 (0.5)	121 (0.8)		11 (0.3)	187 (1.0)	

Data are *n* (%) unless otherwise indicated.

[a] Fragile X, Klinefelter, Laurence-Moon-Bardet-Biedl, Down, Noonan, Prader-Willi, Silver Russell, and Turner

[b] Including brain tumors (malignant and benign).

SES, socioeconomic status.
