# Supplementary material for: Association of childhood obesity with risk of early all-cause and cause-specific mortality: A Swedish prospective cohort study
Source: PLoS Med. 2020 Mar 18;17(3):e1003078. doi: 10.1371/journal.pmed.1003078 (PMC7080224; doi:10.1371/journal.pmed.1003078)
Supplement: S2 Table — (PDF) [file pmed.1003078.s003.pdf]

S2 Table. Primary cause-specific mortality.

**Cause of death**	**Childhood obesity cohort**		**Comparison group**	
	n	%	n	%
**Endogenous causes**				
Cancer	4	10.3	7	10.8
Infections	4	10.3	3	4.6
Endocrine causes	3	7.7	2	3.1
Other[a]	4	10.3	5	7.7
**Injuries and external causes**				
Transportation accident	4	10.3	6	9.2
Homicide	2	5.1	5	7.7
Other[b]	1	2.5	3	4.6
**Suicide and self-harm**				
Suicide	3	7.7	19	29.2
Self-harm with unintentional- or undetermined intent	13	33.3	12	18.5
**Unknown cause**[c]	1	2.5	3	4.6
**Total deceased**	**39**	**100**	**65**	**100**

[a] Including e.g. pulmonary embolism and congenital risk factors.

[b] Including e.g. war action and drowning.

[c] Including e.g. death abroad and declining rates of autopsy.
