# Supplementary material for: Association of childhood obesity with risk of early all-cause and cause-specific mortality: A Swedish prospective cohort study
Source: PLoS Med. 2020 Mar 18;17(3):e1003078. doi: 10.1371/journal.pmed.1003078 (PMC7080224; doi:10.1371/journal.pmed.1003078)
Supplement: S1 Text — (PDF) [file pmed.1003078.s004.pdf]

Translation of relevant parts of the study analysis plan and general methods:

Study analysis plan – Obesity in childhood and risk of premature death

This study is part of a larger epidemiological analysis, using data from our national database with information from several population-based registers.

Background: Obesity reduces life expectancy significantly. Paediatric obesity is associated with increased risk for premature death from middle age, but whether the risk is increased already in young adulthood is unknown.

Specific aims:

- To investigate if young adults, treated for obesity in childhood, have an increased risk of premature death compared with a matched population-based comparison group.
- To study if the cause-specific mortality differs between the groups.
- To investigate if sex, ethnicity, and parental socioeconomic status (SES) affect the risk of premature death in the defined cohort.
- To study if treatment efficacy and degree of obesity are associated with the risk of premature death in the childhood obesity cohort?

Method:

- Observational prospective cohort study (Data acquisition described under "General methods")
- Main outcome is death
- Main exposure is obesity in childhood
- Covariates are among others: SES, BMI SDS and age at treatment initiation ethnicity, sex

Inclusion criteria:

- Registered in BORIS between 3-17.9 years of age
- Alive and living in Sweden at start of follow-up

Follow-up start:

- 18 years of age

Follow-up ends:

- Date of death. Acquired from the cause of death register.
- Date of emigration. Acquired from the Swedish Total Population Register
- End of follow-up, i.e. December 31st, 2017

Statistical analysis plan:

- Descriptive statistics of all participant characteristics presented as n (%), or mean (SD), or median (IQR).
- Crude and adjusted Cox proportional model to calculate mortality rate ratio, 95% confidence intervals and p-values. Adjusted models should control for group (childhood obesity or comparison group), sex, Nordic origin and parental SES.
- Kaplan Meier curve to analyse probability of survival between the groups.

- Sensitivity analyses should be carried out excluding individuals with genetic syndromes and tumors in childhood.
- Secondary analyses in the childhood obesity cohort to investigate the effect of the degree of BMI SDS at obesity treatment initiation, as well as age at start of treatment.

General methods

The Swedish Childhood Obesity Treatment Register (BORIS) was initiated in 2005 and is a prospective register of children and adolescents undergoing obesity treatment. BORIS contains data from treatment visits, including anthropometrical, biochemical data, and results from physiological investigation (e.g. blood pressure and cardiorespiratory fitness).

Data protection, archiving and ethical considerations

Using the Swedish identity number, unique to each individual and provided by the BORIS-administrator, data were linked and collected from several other registers by Statistics Sweden and The National Board of Health and Welfare. After the linkage, when the researcher retrieves the data, the Swedish identity number has been replaced by a key code. Thus, there is no possibility for the researcher to go back and identify subjects in the data itself, nor via the BORIS-administrator. Results will only be presented on an aggregated level, and therefore, it is not possible to trace results back to a specific individual. Statistics Sweden and The National Board of Health and Welfare have been asked to save the code key for coming years to enable additional data extraction.

Data acquisition

18 000 children and adolescents who were registered in BORIS in 2016 participate in the latest register linkage. Among the subjects in BORIS, 7200 are at the time of follow-up (dec 2017) above 18 years. A contemporaneous comparison group from the Total Population Register was historically (year of entrance in BORIS) matched with a ratio of 1:5 by sex, year of birth, and living area. In addition are biological and adoptive parental data collected, both for the subjects in BORIS and for the matched comparison group. We have linked them with the Swedish Total Population Register, the Swedish medical birth register, the medical drug register, the inpatient and outpatient register, the cause of death register, the longitudinal integration database for health insurance and labour market studies (LISA), and the national diabetes register (NDR and SWEDIABKIDS).

Information about consent from study subjects

All healthcare providers reporting data to BORIS follow the current legislation on data collection for national quality registers and the newly adapted Genereal Data Protection Regulation, GDPR. Parents of children have been informed about BORIS before registration. Collecting data in national quality registers require consent to the extent that the care provider is obligated to give written information to the guardians. The patient information should contain information about the register, what will be registered, how the information is handled, confidentiality, security, access and rights to request information, corrections, and removal of data. Most common is that information is mailed to the family together with the remittance to the doctor. In BORIS, this information is currently available in five languages (Swedish, English, Spanish, Arabic and Somali).

Ethical decision

The study was approved by the regional Ethics Committee in Stockholm, Sweden (No. 2016/922-31/1).
