# Supplementary material for: Association of childhood obesity with risk of early all-cause and cause-specific mortality: A Swedish prospective cohort study
Source: PLoS Med. 2020 Mar 18;17(3):e1003078. doi: 10.1371/journal.pmed.1003078 (PMC7080224; doi:10.1371/journal.pmed.1003078)
Supplement: S2 Text — (PDF) [file pmed.1003078.s005.pdf]

Emilia Hagman
Uppdat: 23-04-2016

Hur är fetma bland barn och ungdomar kopplat till användning av mediciner, allmän sjuklighet och psykosocial missanpassning i ung vuxen ålder?

Introduktion

Barnfetma är en sjukdom som leder till ökad sjuklighet[1] och psykosocial missanpassning[2,3] i vuxenåldern och en kraftigt ökad risk för förtidig död[4]. Den ökade dödligheten beror bland annat på ökad insjuknande i cancer men framförallt är det hjärt-kärlsjukdomar associerade till det metabola syndromet som dominerar. Komorbiditet som insulinresistens och högt blodtryck är tidiga konsekvenser av fetma som kan ses redan hos yngre barn[5,6]. Hur stor total andel av barn och ungdomar med fetma som har sjuklighet relaterad till sin fetma är dock oklart.

Studier har visat att fetma som är bestående under barndomen predisponerar för sämre psykosocial hälsa som vuxen. Redan 1993 visade en amerikansk studie hur unga vuxnas psykosociala hälsa försämrades om de haft fetma som tonåringar. Konsekvenser var mest dramatiska hos kvinnor där de såg att 50 % lägre frekvens var gifta, 50 % lägre frekvens hade slutfört college, de hade 40 % lägre inkomst och 2,5 gånger fler levde under existensminimum jämfört med kontrollgrupp[2]. Vidare finns det beskrivet att fetma som bara varit under barndomen har en liten påverkan på den unga vuxnas psykosociala hälsa. Om fetman däremot har varit bestående genom hela uppväxten så visade det sig framförallt påverka möjligheten till arbete och nära relationer[3].

Huruvida det finns riskfaktorer eller framgångsfaktorer som påverkar framtida psykosocial och fysiologisk hälsa bland barn med fetma är inte klarlagt. De studier som finns publicerade är ofta baserade på små material med begränsad representativitet. Vi har ett kvalitetsregister[7], som gör det möjligt att med data från ett stort antal barn kunna besvara ett flertal viktiga frågeställningar.

Barnobesitasregistret i Sverige - BORIS - är det nationella kvalitetsregistret för de barn som behandlas för sin övervikt eller fetma. Det grundläggande syftet med registret är att långsiktigt följa behandling av barnfetma i landet för att kvalitetssäkra vården lokalt gentemot hur det ser ut i Sverige totalt. Ett syfte är att öka kunskapen om olika behandlingsformer. Ett annat grundläggande syfte är att det också ska tillhandahålla enkla arbetsinstrument för lokal kvalitetsförbättring av barnfetmavården.

Hur fetma hos barn och tonåringar påverkar individens sjuklighet och social anpassning i vuxna år är inte utrett och därför är ett av huvudsyftena med denna registerstudie att följa upp samt kartlägga hur fetma hos barn och tonåringar påverkar individen i vuxen ålder. Vi har noterat i BORIS att många barn har förhöjda fasteblodsocker[6,8,9] och att även en störd blodtrycksreglering ofta föreligger[5,10]. Vi vet dock inte i vilken utsträckning detta är relaterat till sjuklighet som unga vuxna.

Vi har också observerat i BORIS att många barn har förhöjda blodtryck och att även barn har förhöjda blodlipider vilket vi redovisat i våra årsredovisningar. Det är däremot inte registrerat att någon har farmakologisk behandling för dessa tillstånd. Detta kan ha flera förklaringar, dels kan indikation för behandling saknas pga att nivåerna normaliseras utan att det registrerats i BORIS, dels kan barnen ha fått behandling utan att detta registrerats. En enstaka observation är inte heller en indikation för behandling och det går således inte att utesluta att en adekvat uppföljning saknas i vissa fall.

Syfte

Huvudfrågeställningen vid analysen är vilka faktorer som är mest betydelsefulla som prediktorer för framtida sjuklighet och försämrad psykosocial anpassning men genom att jämföra med en kontrollgrupp kan vi också få en bild av hur denna utsatta patientgrupp ser ut som unga vuxna mer generellt.

Specifika frågeställningar i relation till huvudsyftet

- Hur påverkar graden av fetma risken för framtida metabol sjuklighet, så som diabetes, högt blodtryck och höga blodfetter?
- Har barn som lider av fetma och samsjuklighet (förhöjt blodtryck, förhöjda levervärden, rubbade blodfetter, etc) ökad sjuklighet som vuxen?
- Hur stor är risken att barn med fetma genomgår viktminskningskirurgi i vuxen ålder?
- Har barn som lider av fetma ökad risk att få maligna tumörer i tidig vuxen ålder?
- Har de barn som lider av fetma ökad risk för förtidig död redan i unga vuxna år?
- Har de som föds små för åldern (SGA) och utvecklar fetma ökad risk för metabol sjuklighet som barn/tonåringar respektive i vuxen ålder jämfört med de som är födda normala för åldern (AGA) och utvecklat fetma?
- Erhåller barn med fetma i Sverige en lägre utbildningsnivå och/eller lägre lön för samma utbildningsnivå?
- Hur stor är kostnaden för samhället för läkemedel och sjukvård kopplat till barnfetma och dess relaterade sjuklighet?

Ett annat syfte är att se om barn har behandling för hyperlipidemi, typ2 diabetes och hypertoni utan att detta är registrerat i BORIS. I anslutning till detta ska vi också undersöka om det finns något samband mellan registrerade förhöjda blodtryck, hyperlipidemi, förhöjda blodsocker och farmakologisk behandling av föräldrar för sådana tillstånd.

För samtliga frågeställningar kan vi både jämföra exponering inom BORIS-registret, men även jämföra med en matchad grupp från normalpopulationen. Vidare kan vi även justera för civilstånd, föräldrarnas sjuklighet och socioekonomiska situation (utbildning och inkomst).

Metod

Samtliga individer i BORIS som per 31 december 2015 var över 5 års ålder kommer att inkluderas i denna studie, n=15 000. Dessa ska följas upp i en observationell prospektiv kohortstudie. Hela kohortens sjuklighet och sociala anpassning ska analyseras. Data analyseras dels utifrån data från BORIS, dels utifrån jämförelse med kontrollgrupp som erhålls från SCB. Fem kontroller per fall kommer att skapas n=75 000. De ska vara matchade på födelseår, kön och bostadsområde. Utöver detta kommer information om föräldrarna att inhämtas (n=((75' + 15')*2)=180 000). Detta för att kunna utvärdera den socioekonomiska situationen och eventuell benägenhet för sjukdomar (diabetes etc).

Följande register kommer (1)individer från BORIS, (2) dess kontroller, samt (3) deras föräldrar att länkas till:

- Longitudinell integrationsdatabas för sjukförsäkrings- och arbetsmarknadsstudier **(LISA)** där finns samtliga personer 16 år och däröver, som varit folkbokförda i Sverige den 31/12 respektive år. Eftersom det är en longitudinell databas kan man över tiden följa individens övergångar mellan exempelvis perioder med förvärvsarbete, arbetslöshet och sjukdom kopplade till ett stort antal bakgrundsvariabler. I databasen finns kopplingsmöjligheter till ytterligare ett antal exponeringar såsom familj och arbetsställe. Dessutom innehåller LISA även mera detaljerade data om sjuk-, föräldra- och arbetslöshetsförsäkringen för individen.
- **Medicinska Födelseregistret** innehåller händelser och utfall för kvinnor och barn under graviditet, förlossning och nyföddhetsperioden.

- **Patientregistret** innehåller alla slutenvårdstillfällen men även öppenvård sedan 2002.
- **Läkemedelsregistret** innehåller alla uthämtade läkemedel från 2005. Med hjälp av detta register kan vi se vilka som får behandling för bl a högt blodtryck, diabetes, höga blodfetter.
- **Dödsorsaksregistret** innehåller datum och orsak för död.
- Från Nationella diabetes registret **(NDR)** hämtas information om patienterna fått diabetes.
- **SWEDIABKIDS** är det nationella kvalitetsregistret för barn med diabetes. Här återfinns behandlingsformer samt andra viktiga parametrar (ex blodsocker, HbA1c, längd och vikt).
- Från FLERGENERATIONSREGISTRET kommer uppgifterna om föräldrar och om barnet varit adopterat. Detta är viktigt när vi tittat på ärftliga faktorer såväl som familjesituation.

All exporterad data är redan kodad vid exportering från BORIS. SCB och Socialstyrelsen ombeds att lämna omkodad data tillbaka till forskaren. Forskaren kommer således aldrig i kontakt med vare sig namn på patienten eller dess personnummer. Den kodade datan kommer att lagras på server tillhörande Karolinska Institutet och kommer att behandlas i en databas. Resultaten kommer enbart att redovisas på aggregerad nivå.

Betydelse

Det saknas uppföljningar från Europa på hur barn med fetma klarar sig psykosocialt jämfört med en matchad kontrollgrupp. Detta behövs för att avgöra i vilken utsträckning barn med fetma utgör en riskgrupp för framtida missanpassning vilket i så fall kan påverka behovet av särskilt stöd för denna grupp.
Det är en brist på longitudinella uppföljningsstudier där det prediktiva värdet av riskmarkörer och framgångsfaktorer hos barn och ungdomar med fetma studerats. Det medför att man idag inte kan använda dessa riskmarkörer för att bestämma hur intensiv behandling bör vara för barn och ungdomar med fetma. Antidiabetika, blodtrycks- och kolesterolsänkande mediciner används i regel inte. Å andra sidan är det heller inte känt om det skulle innebära någon långsiktig fördel för dessa barn och ungdomar att använda mer läkemedel.
Vi kan bidra till att identifiera vid vilken ålder det av medicinska skäl finns anledning att överväga att farmakologiskt behandla de riskmarkörer för framtida sjuklighet som barn och ungdomar med fetma kan ha.
Vidare kan studierna underlätta identifikationen av högriskindivider för tidig utveckling av fetmarelaterad sjuklighet, social missanpassning, det vill säga individer som behöver särskilt intensiv behandling, med beteendemodifiering, läkemedel och fetmakirurgi.
Slutligen kan studierna visa om graden av framgång i behandling av fetma i barndomen påverkar sjuklighet långsiktigt vilket också är av betydelse för hur mycket barnsjukvården ska fokusera på sådan behandling. Idag har endast 10-20% av barn med fetma behandling och flera barnkliniker tar inte emot barn med fetma.

Preliminära resultat och framtida utsikter

År 2014 genomförde vi en första länkning av data från BORIS-registret. I det datauttaget tog vi endast med de individer som hade fyllt 18 år per 31 december 2013 för att kunna utvärdera effekterna av fetma i barndomen till vuxna år (n=3300). Länkningen blev mycket lyckad.
Denna länkning har så här långt resulterat i två artiklar som är inskickade för publikation till vetenskapliga tidskrifter:
Den ena där sannolikheten att ta studenten utvärderats för individer som behandlats för fetma i jämförelse med en grupp matchat från normalpopulation. Av de barn som led av fetma avslutade 55,4% gymnasiet, jämfört med 76,2% i kontrollgruppen.
Den andra där förhöjda fasteblodsockers prediktiva värde på insjuknande i typ 2 diabetes (T2D) utvärderats. Vi använde diabetesläkemedel som markör för T2D. Barn med fetma i Sverige hade en markant ökad risk att få diabetesläkemedel i tidig vuxen ålder i jämförelse med en

populationsbaserad kontrollgrupp, oberoende av kön och etnicitet. Förhöjt fasteblodsocker definierat av WHO (cut-off 6.1 mmol/L) förutspådde framtida användning av T2D-medicinering hos vuxna. Däremot, det sockerintervall som ADA definierat (5.6-6.1 mmol/L), ökade inte användningen av T2D-medicinering hos unga vuxna mer än barnfetma i sig.

Utöver dessa två artiklar har även kostnad för läkemedelsanvändning utvärderats av två studenter från Handelshögskolan i deras magisteruppsats. Vidare har vi gjort analyser på risken för antihypertensiva läkemedel, tumörinsjuknande, viktminskningskirurgi och död. Dock hade vi i den länkningen begränsad power för att besvara dessa frågeställningar. I en ny större länkning är vi förvissade om att tillräcklig power finns eftersom antalet individer från BORIS som blivit vuxna har ökat till ca 5400 samtidigt som uppföljningstiden för de som blivit vuxna är längre.

I länkningen som gjordes 2014 har vi saknat information om föräldrarna. Därför kommer vi i den nya länkningen att begära ut data även från dem. Vidare har vi inte haft data på de individer som varit under 18 år och därmed inte kunnat utvärdera vare sig sjuklighet under barnåren till följd av fetma eller deras sociala anpassning. Denna ytterligare data kommer att öka såväl kvalitén som förståelsen för denna patientgrupps förutsättningar, både under barndomen och framtida aspekter.

Referenser

1. Daniels SR. Complications of obesity in children and adolescents. *Int J Obes (Lond).* 2009;33 Suppl 1:S60-65.
2. Gortmaker SL, Must A, Perrin JM, Sobol AM, Dietz WH. Social and economic consequences of overweight in adolescence and young adulthood. *The New England journal of medicine.* 1993;329(14):1008-1012.
3. Viner RM, Cole TJ. Adult socioeconomic, educational, social, and psychological outcomes of childhood obesity: a national birth cohort study. *BMJ.* 2005;330(7504):1354.
4. Fontaine KR, Redden DT, Wang C, Westfall AO, Allison DB. Years of life lost due to obesity. *JAMA : the journal of the American Medical Association.* 2003;289(2):187-193.
5. Westerstahl M, Hedvall Kallerman P, Hagman E, Ek AE, Rossner SM, Marcus C. Nocturnal blood pressure non-dipping is prevalent in severely obese, prepubertal and early pubertal children. *Acta Paediatr.* 2014;103(2):225-230.
6. Hagman E, Reinehr T, Kowalski J, Ekbom A, Marcus C, Holl RW. Impaired fasting glucose prevalence in two nationwide cohorts of obese children and adolescents. *Int J Obes (Lond).* 2014;38(1):40-45.
7. BORIS. The National Register for Childhood Obesity Treatment www.e-boris.se. www.e-boris.se.
8. Ek AE, Rossner SM, Hagman E, Marcus C. High prevalence of prediabetes in a Swedish cohort of severely obese children. *Pediatric diabetes.* 2015;16(2):117-128.
9. Hagman E, Arani PI, Fischer M, et al. Blood sugar levels are higher in obese young children in Sweden than in Poland. *Acta Paediatr.* 2014;103(11):1174-1178.
10. Westerstahl M, Marcus C. Association between nocturnal blood pressure dipping and insulin metabolism in obese adolescents. *Int J Obes (Lond).* 2010;34(3):472-477.
